# Anticoagulation practices in venous occlusive disease after stent placement

**DOI:** 10.1186/s42155-026-00763-y

**Published:** 2026-10-09

**Authors:** Omar Ayman, Olivia Antonescu, Warren A. Campbell, Hyeonjeong Lim, Eunjee Lee, Jeffrey F. B. Chick, David Shin, Mina S. Makary

**Affiliations:** 1 Division of Vascular and Interventional Radiology, Department of Radiology, The Ohio State University Wexner Medical Center, Columbus, OH USA; 2 Division of Vascular and Interventional Radiology, Department of Radiology, University of Virginia, Charlottesville, VA USA; 3 Department of Information and Statistics, Chungnam National University, Daejeon, South Korea; 4 Division of Vascular and Interventional Radiology, Department of Radiology, University of Southern California, Los Angeles, CA USA

**Keywords:** Survey, Anticoagulation, Antithrombotic, Antiplatelet, Veno-occlusive disease, Venous stent anticoagulation

## Abstract

**Purpose:**

Characterize physician anticoagulation practices after stent placement in patients with venous occlusive disease.

**Materials and methods:**

Survey-based analysis was conducted between January 2023 and April 2023 (for 109 days) evaluating practices of post venous stent placement anticoagulation regimens. A survey investigated provider demographics, agent selection, dosing, duration, and patient factors taken in agent choice. The survey was distributed through email lists and promoted on social media and limited users to one response. Questions stratified for both superior vena cava (SVC) and upper extremity and inferior vena cava (IVC) and lower extremity interventions. Descriptive statistics determined the significance of the regimen utilization.

**Results:**

One hundred twenty-nine respondents participated in the study, with interventional radiologists constituting 94%. The preferred regimen for SVC interventions was direct oral anticoagulants (DOAC) and single antiplatelet therapy, accounting for 39/117 of respondents. For IVC interventions, low molecular weight heparin (LMWH) and single antiplatelet therapy was the regimen of choice representing 12/38 of preferences. SVC intervention therapy duration was most common to be lifetime for aspirin (10; 43%), 3 months for non-ASA antiplatelet agents (23; 52%), and 6 months for anticoagulants (32; 44%). For IVC therapies, the trend of drug duration for aspirin was most common to be lifetime (6;40%), for non-ASA antiplatelets was 3 months (10; 53%), and for anticoagulants tied for 6 and 12 months (7; 30% each). Eight risk factors affected post-intervention anticoagulation selection.

**Conclusion:**

This work highlights the most common post-venous stenting anticoagulation regimens currently in clinical practice.

**Supplementary Information:**

The online version contains supplementary material available at https://doi.org/10.1186/s42155-026-00763-y.

## Introduction

Endovascular venous reconstruction procedures have made notable advancements in addressing deep venous disease leading to venous obstruction and the symptomatic sequelae of chronic venous insufficiency including swelling, limb edema, facial swelling, and overall patient discomfort [1, 2]. The treatment of venous occlusive disease (VOD) involving venous stenosis, compression, fibrotic changes, or residual organized thrombus has increasingly been treated with venous stenting to improve patient quality of life [3]. The most common concern with stent placement is long-term patency with high rates of post-procedural in-stent restenosis and/or stent thrombosis, with rates that may be as high as 28% after 1 year and 56% after 5 years [4]. However, studies including randomized trials, cohort studies, and meta-analysis reviews show that stenting does improve venous patency and quality of life metrics in patients with VOD when compared to venous angioplasty and medical management alone [3, 5–7]. Thus, the Society of Interventional Radiology (SIR) recommends venous stent placement in patients with chronic iliofemoral venous obstruction [4].

Despite a growing consensus for the implementation of venous stents in VOD, there is no established standard for prescribing anticoagulant therapies to patients following such procedures. Common antithrombotic practices include aspirin, single anti-platelet therapy (SAPT), dual-anti-platelet therapy (DAPT), low-molecular weight heparin (LMWH), warfarin, direct oral anticoagulation (DOAC), or a combination of these therapies [8–10]. The duration of therapy can also range from 1 month to continuous anticoagulation after stent placement.

There have been no prospective randomized controlled trials to evaluate the efficacy of stent patency between different regimens and duration of therapy [10]. Small prospective cohorts have demonstrated that DOAC demonstrated similar stent patency and complication rates compared to warfarin and rethrombosis rates on anticoagulation did not vary between 3 and 12 months, with the highest rates of rethrombosis in the first few months [1]. Prior to this study, there was only one prior survey completed investigating an expert consensus in 106 international experts regarding managing after iliac stenting, specifically in May-Thurner syndrome published in 2018 [11]. They developed a consensus that 6–12 months of anticoagulation is appropriate, with LMWH as the preferred agent acutely in the first 6 weeks. Additionally, anticoagulation should be continued life-long with repeated deep venous thrombosis (DVT). The role of antiplatelet agents was not discussed.

The purpose of this study was to survey vascular specialists who manage venous stents in VOD to better understand current clinical practices for antithrombotic therapy. This study provides epidemiological data to better understand what regimens are being implemented such that future studies can investigate the comparative safety and efficacy of these practices.

## Methods

### Study design

The study was reviewed by IRB and was deemed exempt. The study period was conducted between January 2023 and April 2023. The targeted group of physicians were vascular specialists including interventional radiologists, interventional cardiologists, vascular surgeons, hematologists, and vascular medicine physicians. The online survey was distributed virtually by directly emailing attending physicians at institutions with vascular programs (668 programs including integrated vascular surgery, integrated interventional radiology, and fellowships encompassing interventional cardiology, interventional radiology, vascular surgery, vascular medicine and hematology/oncology), social media including LinkedIn (LinkedIn Corporation; Sunnyvale CA), and X, previously known as Twitter (X Corp; Bastrop TX), and online society forums including SIR, Society for Vascular Surgery (SVS), and American Venous Forum (AVF). Survey responses were anonymous, and signed in uses were limited to one submission to minimize duplicate responses.

### Demographics

The characteristics of the study population are described in detail in Table 1. One hundred twenty-nine respondents participated in the study, with interventional radiologists constituting 94%, which limits the generalizability of the survey results. Interventional cardiologists, hematologists, and vascular surgeons constituting 2% each. Practice settings included academic (56; 43.4%), community hospital (69; 53.4%), and outpatient-based labs (OBLs) (4; 3.1%). The time spent in practice settings include urban (82; 64.6%), rural (9; 7.0%), and suburban (38; 29.5%). The country of practice for international responses was an optional free text field in the survey. The survey included how many central vein reconstructions have been performed by survey respondents, with the most common answer 11–50 (34%) and 51–100 (27%) procedures.

**Table 1:** Demographic characteristics of the survey participants

Demographics	Responses (*n*)	(%)
**Specialty**		
Interventional radiology	121	93.80
Interventional cardiology	3	2.33
Hematology	3	2.33
Vascular surgery	2	1.55
Vascular medicine	0	0.00
Total	129	100.00
**Employment**		
Academic medical center	56	43.41
Community hospital/non-academic center	69	53.49
Outpatient-based lab (OBL)	4	3.10
Veterans affairs center (VA)	0	0.00
Pediatric hospital	0	0.00
Total	129	100.00
**Environment**		
Urban	82	63.57
Rural	9	6.98
Suburban	38	29.46
Total	129	100
**Region of practice**		
Northeast	16	12.40
Southeast	37	28.68
Midwest	27	20.93
Southwest	12	9.30
West	16	12.40
International	21	16.28
Total	129	100.00
**Years of practice**		
0–5	49	37.98
6–10	22	17.05
11–20	28	21.71
21–30	23	17.83
> 31	7	5.43
Total	129	100.00
**Number of extremity/thoracic/IVC/ileocaval reconstruction cases**		
0–10	26	20.16
11–50	44	34.11
51–100	35	27.13
101–500	17	13.18
501–1000	6	4.65
> 1000	1	0.78
Total	129	100.00

### Survey design

The electronic survey consisted of 20 questions (appendix) that included free text and multiple-choice questions. The survey was sectioned into four parts: demographics, SVC and upper extremity most preferred anticoagulation regimen, IVC and lower extremity most preferred anticoagulation regimen, and factors that influence the antithrombotic regimen. Each respondent’s management selection was recorded for analysis (Table 2). The frequency of the most common anticoagulation regimens was stratified by stent location and clinical factors that influence regimen selection. The survey investigated the diversity of anticoagulation strategies, exploring variations in anticoagulant choice, dosing regimens, treatment duration, and monitoring practices. Additional factors surveyed include evaluating the impact of patient-specific variables, such as age, comorbidities, and medical history, and on the modification of anticoagulation regimens. Further findings include assessing the extent to which interventionalists identify factors leading to intentional deviations from common protocols.

**Table 2:** Standardized therapeutic dosages for survey

Medication	Dose	Units	Frequency
Aspirin	81	mg	Daily
Clopidogrel	75	mg	Daily
Tirofiban	0.2–0.25	mcg/kg/hr	n/a
Warfarin	2–2.5	INR	daily
UFH	250	U/kg	Q8H
LMWH	1	mg/kg	Q12H
Fondaparinux	1	mg/kg	Q12H
Apixaban	5	mg	Q12H
Rivaroxaban	20	mg	QD

### Statistical analysis

Statistical analysis was performed using Excel (Version 15.0; Microsoft Corp, Redmond, WA). A single proportion test was used to evaluate the preference of specific regimens vs the population to determine if there were statistically significant preferences, with the null hypothesis of the two most selected choices of the 15 possible options listed within the survey represent a threshold percentage (50%). The assumption of independence can be reasonably approximated with a large number of doctors compared to the selection choices. A statistically significant cut-off p value was 0.05, indicating a regimen preference among the respondents. This statistical analysis was performed for a risk factor analysis of factors influencing antithrombotic therapy, with a single proportion test compared to equal weighted selection between every risk factor. A one-sample proportion test was conducted to determine whether each additional factor (54 factors) was associated with a significantly higher rate of regimen change compared to the average proportion of regimen change when any adverse event occurs. Because multiple adverse events can occur at the same time, the observed average proportion (0.279) was used as the reference. The Bonferroni correction was applied for multiple variable hypothesis testing. Lastly, Pareto charts were used to illustrate frequency of common treatment regimens stratified by intervention location.

## Results

### LMWH and SAPT are the most common antithrombotic therapy in IVC and lower extremity VOD

**Fig. 1:**
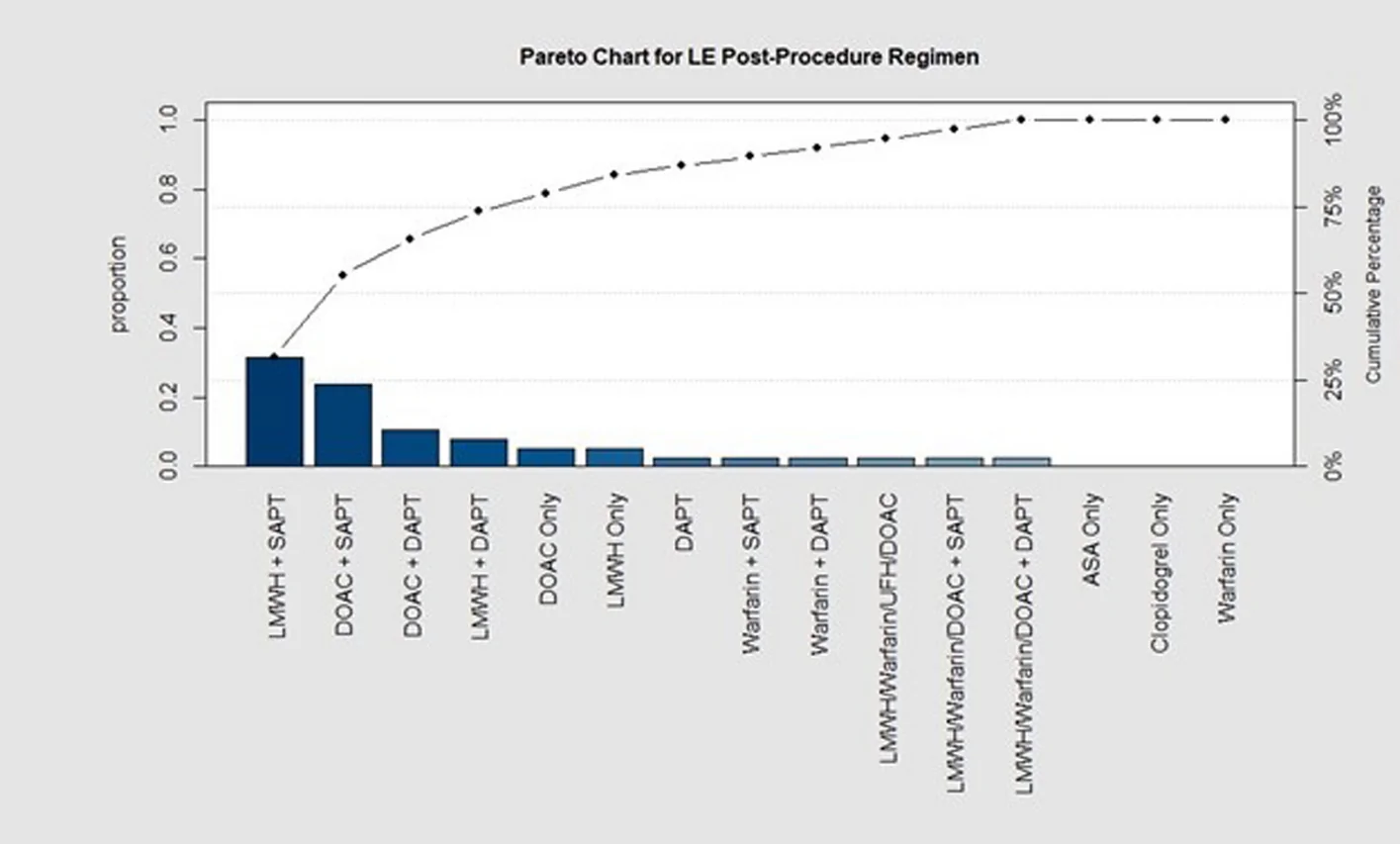
Pareto distribution of antithrombotic regimen selection for IVC and lower extremity venous stents. The selected proportion of each regimen is plotted in order of selection frequency with the cumulative percentage of total respondents representing the top trend line. Low molecular weight heparin (LMWH) + single antiplatelet therapy (SAPT) is the most common regimen, with the top five regimens representing 80% of antithrombotic regimens

Physician antithrombotic therapy preferences following venous stent placement in the IVC and lower extremities were surveyed demonstrating 15 unique therapeutic combinations used in their clinical practice (Table 2). The proportion of their responses were aggregated into a Pareto chart to organize the frequency distribution (Fig. 1). The most common practice was LMWH + SAPT consisting of 31% of respondents. The next most common selection was DOAC and SAPT representing 23% of respondents. These two therapeutic selections did not significantly represent the majority (< 50%, *p* > 0.05) but were the two predominant choices among respondents. Similarly, the cumulative ratio indicates that the top six regimens constitute 80% of the total. Of note, the most common adjunct medication, if not already prescribed, was the addition of a statin medication (11%).

### DOAC and SAPT are the most common antithrombotic therapy in SVC and upper extremity VOD

**Fig. 2:**
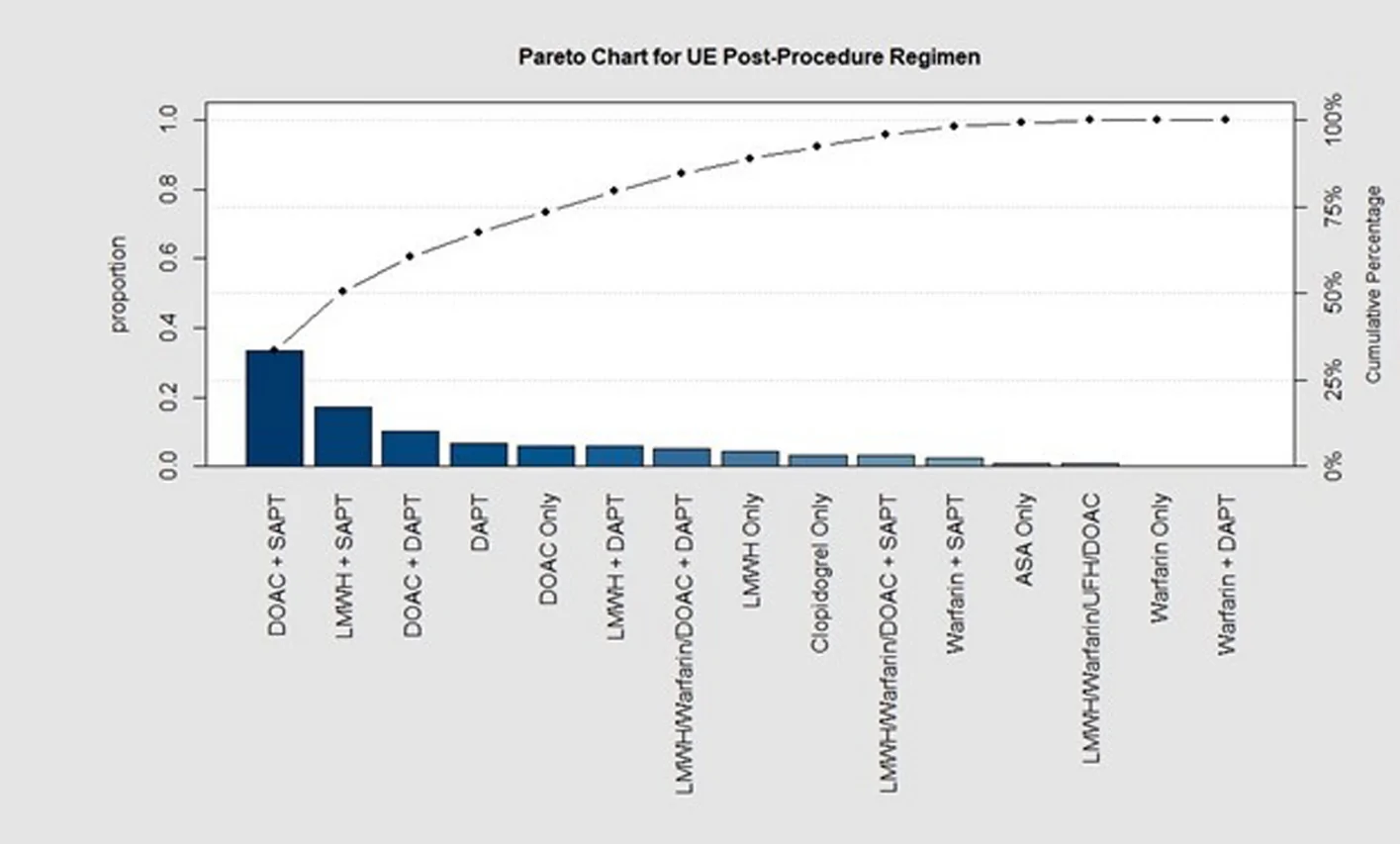
Pareto distribution of antithrombotic regimen selection for SVC and upper extremity venous stents. The selected proportion of each regimen is plotted in order of selection frequency with the cumulative percentage of total respondents representing the top trend line. Direct oral anticoagulants (DOACs) + single antiplatelet therapy (SAPT) is the most common regimen, with the top six regimens representing 80% of antithrombotic regimens

Respondents were surveyed to select the same 15 unique therapeutic combinations used in their SVC and upper extremity VOD clinical practice (Table 2), and findings were aggregated in a Pareto chart (Fig. 2). The most common practice was DOAC + SAPT consisting of 33% of respondents followed by LMWH and SAPT representing 17% of respondents. These two therapeutic selections did not significantly represent the majority (< 50%, *p* > 0.05) but were the two predominant choices among respondents. When comparing the therapeutic combination preferences between upper and lower extremities, DOAC + SAPT was selected 9% more for the upper extremities, where LMWH + SAPT was selected 20% more frequently. The top six regimens for both the upper and lower extremities, as per the accumulated ratio, collectively represent 80% of the total.

### Unique patient risk factors influence antithrombotic regimen

The survey assessed whether the population proportion of regimen change attributed to each factor exceeded the average proportion of regimen change observed in the presence of adverse events (Table 3). Eight variables were significant in selection of upper extremity post-intervention anticoagulation regimens: concurrent anticoagulation, history of deep venous thrombosis occurrence elsewhere, renal insufficiency, hypercoagulable state of malignancy, intracranial metastatic disease, antiphospholipid syndrome, Factor V Leiden mutation, and cost of medication. For lower extremity interventions, the cost of medications was the only significant variable identified (Table 3).

**Table 3:** Influence of common risk factors on antithrombotic regimen in the upper and lower extremity. There were fifty-four factors surveyed among respondents, and with the possibility of each individual factor changing management, an average proportion of 0.279 was utilized. One single proportion test indicates a significant risk factor (*p* < 0.05) to change patient management. Significant variables for the upper or lower extremities were highlighted in bold

Risk factors	Upper extremity (*p* value)	Lower extremity (*p* value)
**Already on anticoagulant therapy (A. Fib, LVAD, etc.)**	**0.000**	0.166
**Yes, I do intraprocedural heparinization with ACT monitoring**	**0.000**	**0.000**
**Intracranial metastatic disease**	**0.000**	0.166
**Cost of medication**	**0.000**	**0.002**
**History of recurrent DVT elsewhere**	**0.000**	0.166
**Antiphospholipid antibody syndrome**	**0.000**	0.083
**Renal insufficiency**	**0.000**	**0.016**
**Factor V Leiden**	**0.000**	0.307
**Hypercoagulable state of malignancy**	**0.000**	0.166
**Malignancy with hemorrhagic propensity (i.e. renal cell carcinoma, anaplastic thyroid carcinoma, choriocarcinoma etc.)**	**0.002**	0.520
**Duplex ultrasound**	**0.003**	**0.026**
**Pregnancy**	**0.010**	0.166
**Poor quality of inflow**	**0.010**	**0.026**
**Prothrombin G20210A (factor II mutation)**	**0.019**	0.520
**Portal hypertension with varices**	**0.019**	0.983
**Protein S and/or C deficiency**	**0.034**	0.307
Poor quality of outflow	0.071	0.062
**Antithrombin III deficiency**	**0.092**	0.788
Yes, malignant venous obstruction alters post procedure anticoagulation regimen	0.109	0.361
**CT Venography**	0.168	**0.010**
Unprovoked DVT	0.203	0.247
Adequate quality of inflow	0.277	0.727
Adequate quality of outflow	0.389	0.727
Hemophilia	0.392	0.520
Hyperhomocysteinemia (MTHFR)	0.392	0.788
Provoked DVTs (transient and chronic)	0.446	0.647
Venous compression syndromes (i.e. May-Thurner syndrome)	0.502	0.788
Pt/INR	0.636	0.727
Limited mobility/sedentary lifestyle	0.726	0.788
Clinical persistence of pain, varicose veins, venous edema, skin pigmentation, inflammation, induration, and ulcer number, duration, and size	0.866	0.727
Clinical worsening of pain, varicose veins, venous edema, skin pigmentation, inflammation, induration, and ulcer number, duration, and size	0.866	0.727
Obesity BMI > 30	0.947	0.788
Sharp/advanced techniques	0.976	0.727
Covered stent (stent graft)	0.976	1.000
Anti-Xa levels	0.980	0.454
Paroxysmal nocturnal hemoglobinuria	0.992	0.983
Clinical improvement of pain, varicose veins, venous edema, skin pigmentation, inflammation, induration, and ulcer number, duration, and size	0.994	0.727
Platelet labs	0.996	0.727
Yes, benign venous obstruction alters post procedure anticoagulation regimen	0.998	0.992
Length of stented segment	0.999	1.000
Congenital atresia of the SVC or IVC	1.000	0.788
Blunt/standard techniques	1.000	0.968
Bare metal stent	1.000	1.000
Type II diabetes mellitus	1.000	1.000
Chronic inflammatory diseases (i.e. Sarcoid, Lupus, Rheumatoid arthritis, Inflammatory bowel disease)	1.000	1.000
Smoking	1.000	0.788
MR venography	1.000	0.968
Hypertension	1.000	1.000
Hyperlipidemia	1.000	1.000
COVID-19	1.000	1.000
Bleeding propensity	1.000	1.000
Other genetic comorbidities	1.000	1.000
Number of stents used	1.000	1.000
D-dimer levels	1.000	1.000

### Aspirin was often prescribed for life, where anticoagulation and non-aspirin anti-platelets were variable between 1 and 12 months

**Fig. 3:**
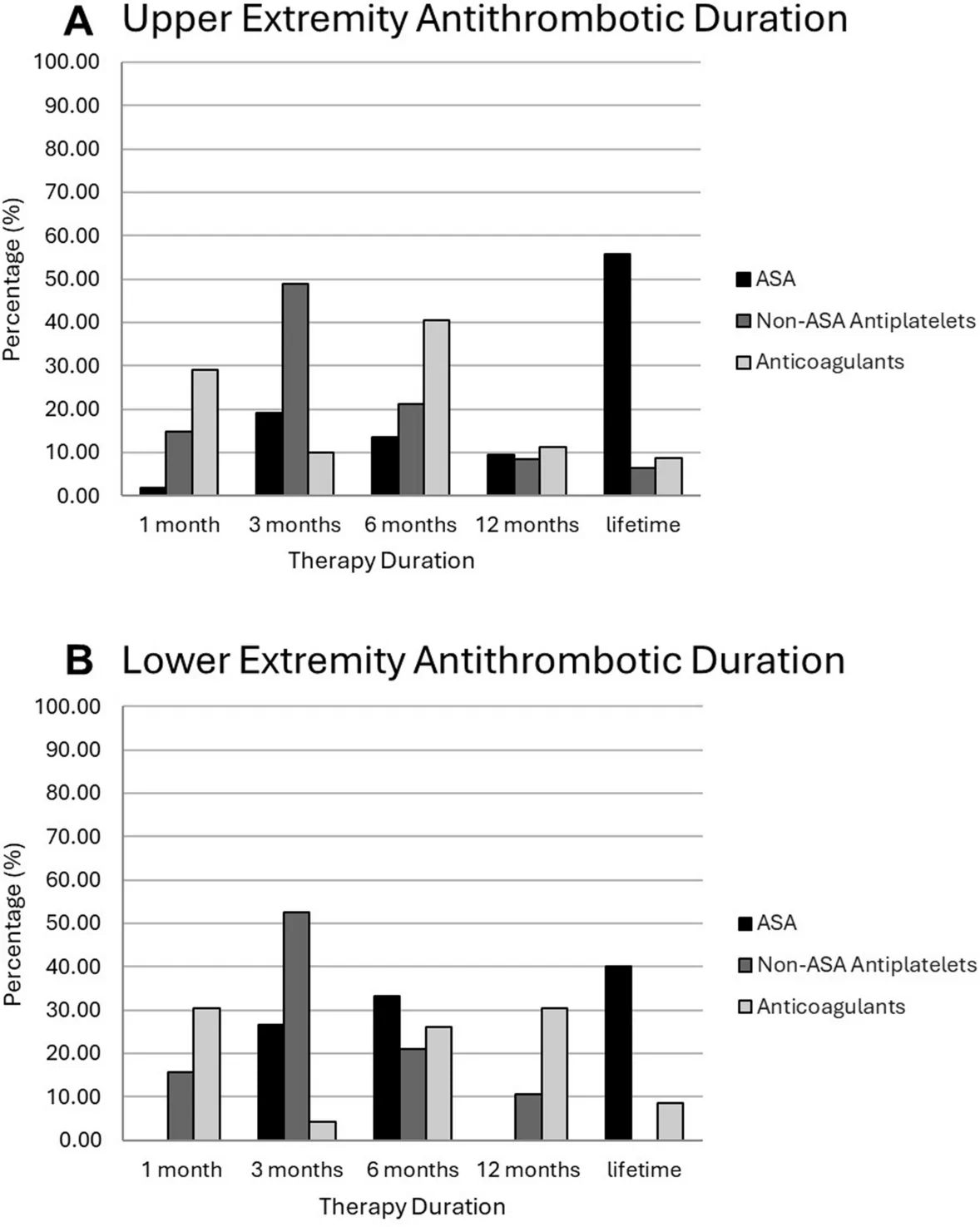
Duration of antithrombotic and anticoagulation therapy post venous stent placement in the upper (**A**) and lower (**B**) extremity. Providers answered survey questions for their preferred anticoagulation and antithrombotic therapy duration after venous stent placement. The responses were categorized by aspirin (ASA), PG2Y12 or Glycoprotein IIb/IIIa inhibitors (non-ASA), and anticoagulation agents including Xa inhibitors, antithrombin mediated anticoagulation, and warfarin (anticoagulation). The survey trend demonstrates that non-ASA is most frequently prescribed for 3 months for the upper and lower extremity. There was a high percentage of patients on ASA, more in the upper extremity than the lower extremity. There was no pattern for anticoagulation in the upper and lower extremity

Between aspirin, clopidogrel/ticagrelor, and anticoagulants such as heparin and DOACS, the recommended duration of treatment varied significantly. In SVC or upper extremity procedures, the prevailing pattern for the duration of aspirin therapy leans towards a lifetime regimen (Fig. 3A). Conversely, for non-aspirin antiplatelets, particularly clopidogrel as the most frequently chosen agent, the predominant trend is 3 months, while anticoagulants are commonly prescribed for a duration of 6 months. For IVC or lower extremity procedures, the most common recommendation (40%) was a lifetime regimen for aspirin (Fig. 3B). In the case of non-aspirin antiplatelets, the favored duration is 3 months, and for anticoagulants, both 6- and 12-month regimens are common.

## Discussion

At the time of this research, there are no prospective studies evaluating the anticoagulation strategies after venous stent placement [10]. Physician antithrombotic therapy preferences following venous stent placement surveyed and analyzed demonstrate similarities in treating upper and lower extremity veins, and highlight variability in antiplatelet duration summarized in Table 4.

**Table 4:** Summary table highlights similarities and differences between upper and lower extremity treatment selection of survey respondents

**Parameter**	**Lower extremity/IVC (**Fig. 1**)**	**Upper extremity/SVC (**Fig. 2**)**
Unique regimens reported	15	15
Most common regimen	LMWH + SAPT (31%)	DOAC + SAPT (33%)
Second most common	DOAC + SAPT (23%)	LMWH + SAPT (17%)
Two leading regimens combined	54%	50%
Modal duration—ASA	Lifetime (~ 40%)	Lifetime (~ 56%)
Modal duration—non-ASA antiplatelet	3 months (~ 53%)	3 months (~ 49%)
Modal duration—anticoagulant	1 month (~ 31%)/12 months (~ 31%)	6 months (~ 41%)

There are retrospective studies that support the use of both anticoagulation and antiplatelet therapy to reduce the risk of acute stent rethrombosis [12–15]. Additionally, there is growing evidence of the role of inflammation in venous thrombosis with significant correlation of increased risk in the setting of systemic infectious inflammatory response, surgery, obesity, and malignancy [16]. While both LMWH and DOAC have been shown to produce similar risks and benefits of bleeding and rethrombosis, there is evidence of the anti-inflammatory role of LMWH which may reduce the risk of acute embolism, particularly after venous stent placement [17].

In IVC and lower extremity interventions, survey results demonstrate that LMWH and SAPT are the most common antitherapeutic regimen, with the other three most popular options consisting of combinations of DOAC and DAPT. This is likely to reflect the current trends in venous stenting literature [18]. There is a clear trend among respondents to risk-stratify patient risk of stent thrombosis focusing on hypercoagulable states such as additional venous thrombosis, renal insufficiency, malignancy, antiphospholipid syndrome, and factor V Leiden. Conversely, hemorrhagic risk was clearly emphasized with consideration of intracranial malignancy.

In the SVC and upper extremities, the overall trend demonstrated more heterogeneity in the selection of agents and consideration of certain risk factors. There is significantly less data focusing on upper extremity venous stent placement in VOD. In non-thrombotic iliac vein lesions, the use of anticoagulation is controversial [19–22]. Similarly, many of these patients with upper extremity VOD also have multiple comorbidities, where anticoagulation is implemented prior to intervention. Therefore, agent selection may be more focused toward general DVT prevention than specific in-stent thrombosis based on patient risk factors. However, in the lower extremity, there is data demonstrating that stent size and location have an impact on therapy durability. Thus, the heterogeneity also likely reflects the lack of data investigating the risks and antithrombotic therapy in the upper extremity.

Furthermore, there is also a lack of consistent evidence in discussing the duration of antithrombotic therapy. Data demonstrates the highest risk of stent re-thrombosis occurs in the acute and subacute setting, supporting the use of anticoagulation [4, 13]. The general trend among respondents was anticoagulants and antiplatelets for 3 to 6 months after stent placement, but there was a general trend to continue aspirin for life. Again, these are likely highly dependent on the individual patient’s risk profile with evidence to suggest that prolonged antiplatelet therapy can improve patency up to 5 years after placement in iliofemoral disease [23].

Prior research suggests the majority of those prescribed aspirin in any combination were advised to continue the regimen indefinitely, while a similar proportion of patients prescribed clopidogrel were instructed to maintain the medication for a 3-month period [11]. The duration of anticoagulant treatment exhibited greater variability, with some patients prescribed for 3 months, others for 6 months, and some indefinitely. Another study recommended lifelong anticoagulation therapy for patients with multiple DVTs and only 6 to 12 months for those with non-thrombotic etiologies [1, 11].

Subsequent research suggests that in the absence of universally accepted guidelines, the available data advocates a potential approach involving anticoagulation within the 3–12 months post-stent placement. This strategy is often combined with a single-agent antiplatelet therapy (such as aspirin 81 mg or clopidogrel) for patients with thrombotic disease lacking a requirement for lifelong anticoagulation [22]. The preference is towards employing a DOAC in the absence of contraindications; otherwise, warfarin is considered [19]. For individuals without a history of multiple deep venous thromboses, current data does not support extending therapy beyond 1 year. Therefore, patients are anticoagulated for 6–12 months, contingent on the quality of venous inflow. Those with multiple deep venous thromboses, additional indications for anticoagulation, or extra risk factors are typically referred for consultation with a hematologist. In such cases, the extension of anticoagulation, potentially for a lifetime, may be considered reasonable [4].

The limitations of this study include a limited sample size for lower extremity management, demographic bias toward newer physicians in practice, and limited international participation. Our survey reflects a limited representation of physician specialties beyond interventional radiology such as vascular medicine and vascular surgery, limiting the generalizability of the results. Depending on the healthcare infrastructure of the country, there may be confounding factors that influence antithrombotic selection beyond optimal medical management. Price of medication was included as an important factor in selection, but there are also other factors not assessed in the survey that may indicate differences in drug selection, duration, and patient compliance. Despite these limitations, this survey is the largest, most recent, and only analysis of current trends in antithrombotic regimen management after venous stents in VOD.

## Conclusion

There is limited clinical data about the optimal antithrombotic regimen after the placement of a venous stent in VOD for the upper and lower extremity. This work provides clinical data about the current preferences of vascular specialists and what factors determine their decision making in altering their clinical plan. While there is a general preference about anticoagulation and antiplatelet therapy acutely after stent placement, the duration and agent selection remain widely variable. This clinical data can provide future justification for antithrombotic selection in future clinical research to develop a formal therapeutic guideline.

## Supplementary Information

Supplementary Material 1.

## Data Availability

Raw data can be made available upon request.
