# Supplementary material for: Anticoagulation practices in venous occlusive disease after stent placement
Source: CVIR Endovasc. 2026 Oct 9;9:129. doi: 10.1186/s42155-026-00763-y (PMC13652999; doi:10.1186/s42155-026-00763-y)
Supplement: Supplementary file 1 [file 42155_2026_763_MOESM1_ESM.pdf]

Survey:

[redacted] Consent to Participate in Research

Study Title:
Questionnaire Surveying Post Procedure Anticoagulation Regimen of Primary Endovascular Superior Vena Cava (SVC) and Iliocaval Reconstructions

Protocol Number:
2022E0990

[redacted]

Purpose: The purpose of this study is to obtain a present view of what current anticoagulation regimens clinicians use post venous reconstruction intervention and what factors deviate from the clinician's standard regimen.

Procedures/Tasks:

An online survey was created to evaluate the current practices of post procedure anticoagulation regimens. The survey also set out to identify what factors changed a patient's anticoagulation therapy. The survey consists of 15 questions that included preselected answer choices and fill-in-the-blank when selecting the "other" option. The survey begins with demographic questions followed by questions regarding anticoagulation therapy and factors that alter the physician's standard regimen. Informed consent is implied by returning the survey to the investigators. We will send the survey out on social media, online forums, and emails to physicians and program coordinators to forward to physicians. When we have our responses collected we will analyze the data and see what the current trends of anticoagulation post procedure therapies currently are and what factors change it.

Participant Rights:

You may refuse to participate in this study without penalty or loss of benefits to which you are otherwise entitled. If you are a student or employee at Ohio State, your decision will not affect your grades or employment status.

If you choose to participate in the study, you may discontinue participation at any time without penalty or loss of benefits. By agreeing to participate, you do not give up any personal legal rights you may have as a participant in this study.

This study has been determined Exempt from IRB review.

Contacts and Questions:

[redacted]

Providing consent

I have read (or someone has read to me) this page and I am aware that I am being asked to participate in a research study. I have had the opportunity to ask questions and have had them answered to my satisfaction. I voluntarily agree to participate in this study. I am not giving up any legal rights by agreeing to participate.

To print or save a copy of this page, select the print button on your web browser.

Please click the button below to proceed and participate in this study. If you do not wish to participate, please close out your browser window.

SURVEY

This survey is a part of an academic collaboration between the Ohio State University's and University of Washington's Interventional Radiology departments, and it is designed to survey the anticoagulation regimen following superior vena cava and iliocaval venous reconstruction as there are currently no definite guidelines on optimal therapy.

Venous reconstruction refers to the process of recanalizing a previously obstructed vein by first balloon-dilating the vessel prior to deploying a stent over the diseased venous segment to maintain venous patency.

This survey is expected to take 5 to 10 minutes to complete. The questionnaire begins with a few demographic and background questions. This is followed by several questions asking what your anticoagulation regimen is and then delving further into what factors affect it and how it changes your management. This survey is completely anonymous.

If you have any questions regarding this study, please contact Olivia Antonsecu, MD (olivia.antonescu@osumc.edu) or Omar Mohamed, MD (omar.mohamed@osumc.edu).

Thank you for your participation in this survey.

Physician Demographics

- **1 What is your specialty?**
  - Interventional Radiology?
  - Interventional Cardiology?
  - Vascular Surgery?
  - Vascular Medicine?
  - Hematology?
  - Other?

- **2 What best describes your practice where you spend the most of your time?**
  - Academic Medical Center
  - Community hospital/Non-academic Center
  - Outpatient- based lab [OBL]
  - Veteran Affairs Center [VA]
  - Pediatric Hospital

- **3 What is the setting you spend most of your time in your practice?**
  - Urban
  - Rural
  - Suburban

- **4 What region is your practice located in?**
    - Northeast
    - Southwest
    - West
    - Southeast
    - Midwest
    - International
        - Free Text

[**203 Map Of Us Divided Into Regions Illustrations & Clip Art - iStock (istockphoto.com)**]

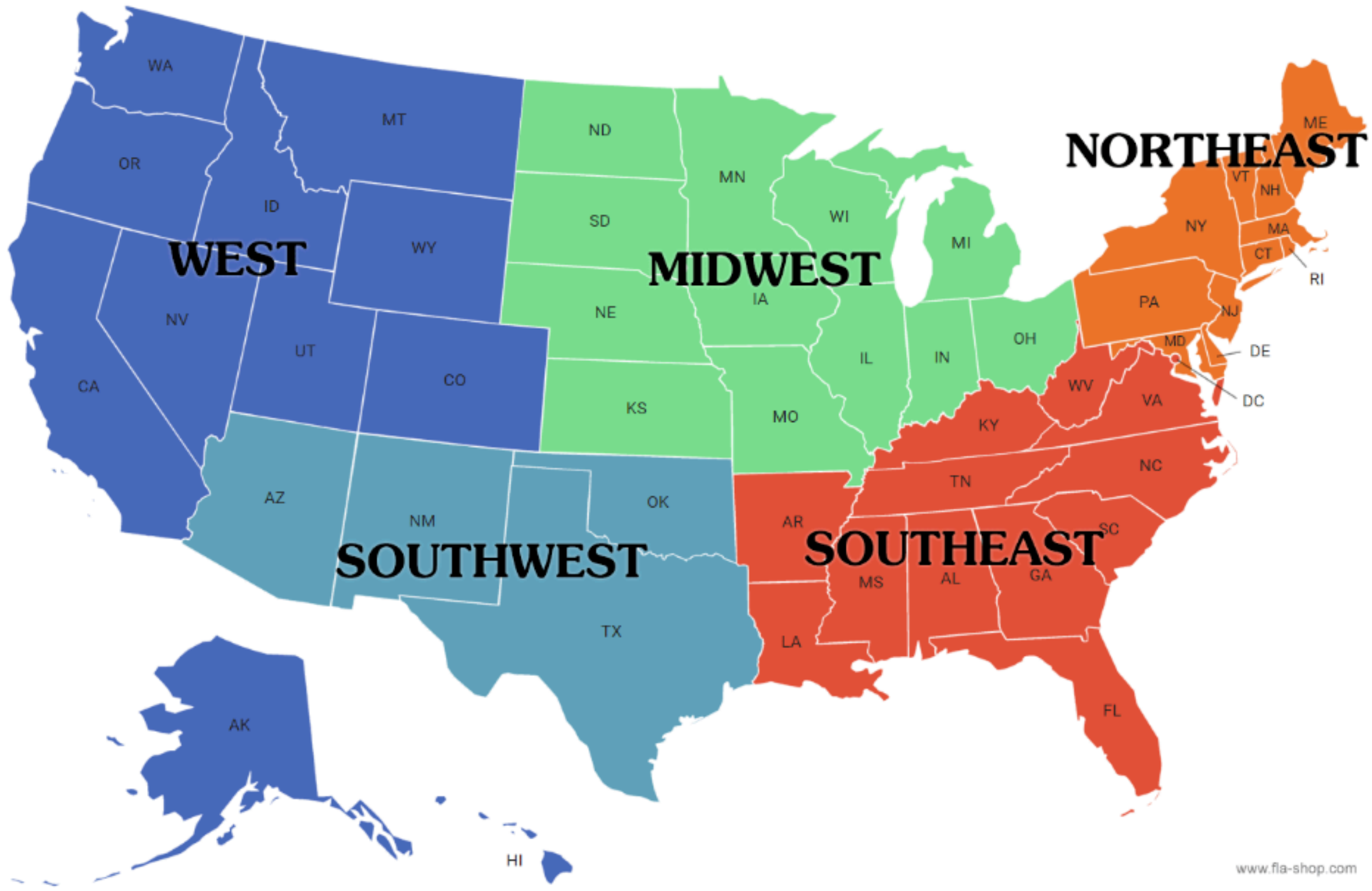

https://www.fla-shop.com/resources/us-regions/

- **5 How many years have you been in practice as an attending?**
    - 0-5
    - 6-10
    - 11-20
    - 21-30
    - >31

- **6 How many Cases of Venous Reconstruction have you approximately performed to date?**

- 0-10
- 11-50
- 51-100
- 101-500
- 501-1000
- >1000

<u>Please answer the following set of questions unique to venous reconstruction of superior vena cava and Iliocaval *thrombotic* obstruction applicable to your procedural experience:</u>

- **7 What is your standard post procedure anticoagulation regimen?**
  - Antiplatelets
    - Aspirin
    - Clopidogrel
    - Prasugrel
    - Ticlopidine
    - Ticagrelor
    - Cangrelor
    - Dipyridamole
    - Cilostazol
    - Eptifibatide
    - Tirofiban
    - Other

    Free text length & dosage (for all choices): _____

  - Anticoagulants
    - Warfarin
    - Unfractionated Heparin
    - Low-molecular Weight Heparin
    - Fondaparinux
    - Dabigatran
    - Argatroban
    - Bivalirudin
    - Apixaban
    - Rivaroxaban
    - Edoxaban
    - Other

    Free text length & dosage: _____

  - Miscellaneous
    - Pentoxifylline
    - Statins

        - Colchicine
        - Venocative supplements
        - Other

    Free text length & dosage: _____

    For the following drug selected: _____ please answer typical regimen dosage and duration:

        - Duration
        - Dosage

- **8 Which presentations of deep venous thrombosis (DVT) alter the choice of anticoagulation?**
    - Provoked DVT (transient and chronic)
    - Unprovoked DVT
    - The presentation of DVT does not alter choice of anticoagulation

- **9 Does the etiology of non-thrombotic occlusions (ie. May-Thurner syndrome, congenital atresia, etc.) alter your standard post-procedure anticoagulation regimen compared to thrombotic occlusions?**
    - No
    - Yes

- **10 Does the etiology of obstruction alter the choice of anticoagulant?**
    - Yes, benign venous obstruction alters post procedure anticoagulation regimen
    - Yes, malignant venous obstruction alters post procedure anticoagulation regimen
    - No, the etiology of venous thrombotic embolism does not alter choice of anticoagulant

- **11 What factors affect your anticoagulation post procedure regimen? Select only the following variables that change your standard regimen:**
    - Age of patient?
        - 0-18
        - 19-65
        - >65
    - Medical Comorbidities?
        - Hypertension
        - Hyperlipidemia
        - Type II Diabetes Melitus
        - Smoking
        - Obesity BMI ≥ 30
        - Pregnancy
        - Already on anticoagulant therapy (A. Fib, LVAD, etc.)

        - History of recurrent DVT *elsewhere*
        - Limited Mobility/Sedentary Lifestyle
        - Renal Insufficiency
        - Portal Hypertension with Varices
        - Hypercoagulable State of Malignancy
        - Malignancy with hemorrhagic propensity (ie. Renal cell carcinoma, Anaplastic thyroid carcinoma, choriocarcinoma etc)
        - Intercranial metastatic disease
        - COVID-19
        - Chronic inflammatory diseases (ie. Sarcoid, Lupus, Rheumatoid arthritis, Inflammatory bowel disease)
        - Other Medical Comorbidities

    - Genetic Comorbidities?
        - Antiphospholipid Antibody Syndrome
        - Factor V Leiden
        - Prothrombin G20210A (Factor II Mutation)
        - Hyperhomocysteinemia (MTHFR)
        - Hemophilia
        - Antithrombin III deficiency
        - Protein S and/or deficiency
        - Paroxysmal nocturnal hemoglobinuria
        - Other Genetic Comorbidities

    - Cost of medication
    - None of the factors above affect my anticoagulation regimen

- **12 Do you perform intraprocedural heparinization with ACT monitoring? *No golden questions***
    - Yes, I do intraprocedural heparinization with ACT monitoring
    - No, I do not do intraprocedural heparinization

- **13 Which of the following procedural techniques and patient clinical outcomes affect your anticoagulation regimen? Select only the following variables that change your standard regimen:**
    - Extent of Reconstruction
        - Length of stented segment
        - Number of stents used
    - Location of Stent
        - Extending the stent below the inguinal ligament
    - Type of Stent Used
        - Bare Metal Stent
        - Covered Stent (Stent Graft)

- Complexity of procedure
    - Sharp/advanced technique
    - Blunt/standard technique
- Robustness of established flow
    - Adequate quality of Inflow
    - Adequate quality of Outflow
    - Poor quality of Inflow
    - Poor quality of Outflow
- Clinical Outcomes
    - Clinical improvement of pain, varicose veins, venous edema, skin pigmentation, inflammation, induration, and ulcer number, duration, and size
    - Clinical persistence of pain, varicose veins, venous edema, skin pigmentation, inflammation, induration, and ulcer number, duration, and size
    - Clinical worsening of pain, varicose veins, venous edema, skin pigmentation, inflammation, induration, and ulcer number, duration, and size
- None of the factors above affect my anticoagulation regimen

- **14 Which of the following ancillary labs or imaging studies do you obtain post-procedure that affect your anticoagulation regimen? Select only the following variables that change your standard regimen:**
    - Platelet Labs
    - Pt/INR
    - D-dimer levels
    - Anti-Xa Levels
    - Duplex Ultrasound
    - CT Venography
    - MR Venography
    - None of the factors above affect my anticoagulation regimen

- ***15 How would the selected factor: "ANSWER FIELD"change treatment? (this question will be posed following the selected factors in questions 8, 9 if answered yes, 10, 11, 13, and 14)***
    - ***Would you change the treatment agent(s)?***
        - ***Yes***
        - ***No***
        - ***If yes, how?***
    - ***Would you change the treatment dosage(s)?***
        - ***Yes***
        - ***No***

    - ***If yes, how?***
  - ***Would you change the treatment duration?***
    - ***Yes***
    - ***No***
    - ***If yes, how?***
  - ***How would you change any other metric of treatment?***
    - ***Insert Text***

[redacted]
